# Engineered Nanomaterials for Environmental and Health Applications

**DOI:** 10.3390/nano15070516

**Published:** 2025-03-29

**Authors:** Lucia Rocco

**Affiliations:** Department of Environmental, Biological and Pharmaceutical Sciences and Technologies (DiSTABiF), University of Campania Luigi Vanvitelli, 81100 Caserta, Italy; lucia.rocco@unicampania.it

In recent decades, the rapid advancement of nanotechnology has led to the introduction of engineered nanomaterials (ENMs) into the environment, whether through deliberate actions or accidental releases. This surge in the use of ENMs has prompted a significant increase in ecotoxicological research focusing on their environmental impact, revealing numerous important concerns that need to be addressed.

However, the deployment of nanotechnology is not without risks; it presents both advantages and drawbacks, particularly regarding human exposure to new nanomaterials. The presence of ENMs in the environment can compromise human safety through various channels. Environmental pollution can lead to unintentional exposure, which may occur in everyday settings or workplaces where these materials are present. Additionally, there is a risk of intentional exposure through specific applications such as nanomedicine, where ENMs are deliberately used for therapeutic purposes.

Conversely, nanotechnology also offers promising applications for environmental remediation, where ENMs are utilized to clean contaminated environments, including soil, water, air, groundwater, and wastewater, a process known as nanoremediation. This innovative approach has the potential to effectively address pollution and restore ecosystems.

Fifteen papers were collected in two editions. Thirteen research articles (eight from the first and five from the second edition) and two reviews (from the first edition) provided interesting contributions about various aspects of ecotoxicity assessments of nanomaterials, including their presence, behavior, fate, and health influence in the environment. Some of them highlighted the potential of certain nanomaterials to mitigate environmental pollution. By bringing together diverse research findings, the two editions of this Special Issue greatly enriched the understanding of both the risks and benefits associated with nanomaterials in ecological and health contexts.

Among the research articles, the study conducted by Vaze et al. highlighted the innovative use of engineered water nanostructures (EWNSs) as an antimicrobial platform that is capable of inactivating human coronavirus 229E (HCoV-229E), which serves as a surrogate for the SARS-CoV-2 virus. This approach offers several advantages over traditional methods of disinfection. One of the key benefits of the EWNS technology is its efficiency in utilizing significantly smaller quantities of active ingredients while still achieving effective virus inactivation. This not only reduces the amount of chemical agents that is needed, it also minimizes potential environmental and health impacts associated with higher concentrations of disinfectants [1].

In their article, Essghaier et al. provided the first evidence for synthesizing silver nanoparticles (AgNPs) using *Scabiosa atropurpurea* subsp. *maritima* (L) fruit extracts. This approach underscores the potential of plant-based extracts in the green synthesis of nanoparticles, offering an environmentally friendly alternative to traditional chemical methods. In addition, the authors assessed the antioxidant, antibacterial, antifungal, and antitoxic effects of the biosynthesized AgNPs against clinical strains, including *Candida* species, highlighting their pharmaceutical and biomedical significance. This research enhances the understanding of the biosynthesis of nanoparticles and their potential biomedical applications, demonstrating the efficacy of plant extracts in generating biologically active nanoparticles [2].

In their contribution, Lionetto et al. demonstrated the efficacy of a new polyethylene terephthalate (PET) model of nanoplastics using a rapid top-down mechanical fragmentation approach. A key finding is the autofluorescence of the label-free PET nanoparticles (NPs), which exhibit fluorescence properties that are comparable to those of Nile red-labeled PET NPs. This property confirms their suitability for biological investigations, as demonstrated through in vitro experiments with *Mytilus galloprovincialis* hemolymphatic cells over a six-hour exposure period. The internalization of label-free NPs into hemocytes suggests that these cells could be primary targets for PET NP effects in mussels. The study evidenced the advantages of using label-free nanoparticles for plastic risk assessments, as they avoid additional toxicity from fluorescent dyes and resist photobleaching, paving the way for more accurate and longer-duration biological studies [3].

Mulder et al., in their article, reported the results of their investigation of the potential genotoxic effects of functionalized gold nanoparticles (GNPs) on Human hepatocellular carcinoma (HepG2) cells. While GNPs offer various benefits, research on their toxicity and influence on human health remains scarce. The authors evaluated both the chronic toxicity and genotoxicity of the functionalized GNPs in these cells. Their findings highlighted the need for further investigation into the genotoxic effects of GNP ligands and other nanoparticle types, particularly regarding the impacts of acute and chronic exposure at sub-toxic concentrations, to better understand their safety profile and implications for human health [4].

Caldera et al. described the development of magnetic nanocomposites for targeted drug delivery, utilizing magnetite/maghemite nanoparticles embedded in a dextrin-based nanosponge. The synergy between iron oxide’s magnetic properties and the controlled release capabilities of the cyclodextrin-based nanosponge (CD-based NS) facilitates effective drug delivery. Magnetite nanoparticles were synthesized through a coprecipitation method, ensuring uniform entrapment in the polymer matrix. Two nanosponge variants were created, using maltodextrin as an alternative to β-CD. The characterization methods included FTIR, TGA, XRD, FESEM, and HRTEM. Finally, the ability of the magnetic NSs to efficiently encapsulate an anticancer drug molecule and release it with controlled kinetics was assessed in vitro, using Doxorubicin as a model drug [5].

Guidi et al. highlighted the potential of a cellulose-based nanosponge (CNS) as an eco-friendly material that is capable of mitigating the genotoxic effects of zinc on the hemocytes of zebra mussels (*Dreissena polymorpha*). This research marks the first demonstration of CNSs’ effectiveness in reducing Zn-induced genotoxicity, suggesting their utility in nanoremediation efforts aimed at improving freshwater ecosystems. By employing both acute toxicity tests and sublethal endpoints, the authors believe that it is possible to gain a comprehensive understanding of the safety and effectiveness of nanomaterials derived from renewable and sustainable sources [6].

Fu et al. studied a novel photoreactive composite, a three-dimensional (3D) core–shell structure combining titanium dioxide (TiO_2_) as the core and crumpled graphene oxide as the shell (TiGC). This composite demonstrated remarkable efficacy in removing and degrading pharmaceuticals and personal care products (PPCPs) in complex aqueous environments. The study quantified reactive oxygen species (ROS) yields, providing insights into the mechanisms underlying the enhanced performance of TiGC. These findings showed the potential of TiGC as a powerful tool for addressing environmental contaminants, particularly in water treatment applications targeting PPCPs [7].

In their study, Sooklert et al. presented the size-dependent immunomodulatory effects of gold nanoparticles (AuNPs), revealing that varying particle sizes could exert both pro-inflammatory and anti-inflammatory effects. The authors believe that the mechanism of action of AuNPs in the reduction and activation of Toll-like receptor 2 (TLR2) pathway signaling remain poorly understood. Additionally, the immune response elicited by AuNPs in conjunction with leptospires is not yet fully characterized, necessitating further investigation. To enhance the therapeutic efficacy of AuNPs for treating inflammation and related disorders, it is crucial to meticulously evaluate all the physicochemical properties of these nanoparticles. Furthermore, the authors recommended conducting additional experiments to analyze the proteins that are produced by pro-inflammatory mRNA genes following treatment, which could provide deeper insights into the immunomodulatory mechanisms at play [8].

This Special Issue also features two reviews. The first one, by Rahim et al., summarized recent advancements in smart nanoformulations and delivery systems that enhance crop protection and nutrition, nanoremediation techniques for polluted soils, and nanosensors for monitoring plant health and food safety. The paper also discussed nanomaterial-based solutions for smart food packaging. Additionally, it emphasized the effects of engineered nanomaterials on soil microbial communities and addressed potential environmental and human health risks associated with their use [9].

The article by Corsi et al. reviewed the behavior and effect of engineered nanomaterials (ENMs) in marine environments, focusing on titanium dioxide (n-TiO_2_) and silver nanoparticles (AgNPs), alongside polymeric nanoparticles like polystyrene (PS), which are often used as nanoplastic representatives. It discussed the interactions of ENMs with both natural and anthropogenic chemicals, leading to eco- and bio-coatings that influence their uptake and toxicity in marine individuals. The article advocated for an ecologically based design strategy to guide the increase in new ENMs, particularly for environmental applications like nanoremediation, ensuring their effectiveness while minimizing risks to marine organisms and humans [10].

In the second edition, the article by Shaban considered anodic aluminum oxide membranes (AAOMs) and their gold-coated variants (AAOM/Au) to create ZnO/AAOM and ZnO/ZnAl_2_O_4_/Au nanoarrays with varying morphologies. The effects of electrodeposition parameters on nanostructure morphology were analyzed via field emission scanning electron microscopy. The ZnO/ZnAl_2_O_4_/Au electrode effectively removed 100% of 20 ppm methylene blue and methyl orange dyes after 50 and 180 min, respectively, following pseudo-second-order kinetics. Additionally, AAOM/Au and ZnO/ZnAl_2_O_4_/Au nanoarrays exhibited pH sensing capabilities, with AAOM/Au showing Nernstian behavior and higher sensitivity compared with ZnO/ZnAl_2_O_4_/Au [11].

In their pioneering study, Guidi et al. explored the efficacy of cross-linked nanocellulose (CNS) combined with a fine-mesh net for treating an environmental matrix laden with various inorganic pollutants, including Zn, Ni, Cu, Fe, Ba, and As. This innovative approach, tested at the laboratory scale, aimed to validate the decontamination efficiency of CNS in a simulated environment. The integration of CNS with the filtering net prevented the direct release of CNS into water during the sludge treatment. To assess the remediation process efficacy, the authors performed a series of ecotoxicity tests and evaluated sublethal biomarker responses in model freshwater species. In particular, the genotoxicity findings demonstrated that the CNS and fine-mesh net combination was both effective and safe for mitigating the harmful effects of contaminated sludge on freshwater ecosystems, marking a significant advancement in environmental remediation strategies [12].

The paper by Essgaier et al. presented the first green synthesis of silver nanoparticles (AgNPs) using the extremophile plant *Aeonium haworthii*. The synthesized AgNPs were characterized through UV-Vis, FTIR, and STM analyses. The study highlighted their antioxidant, antidiabetic, and antimicrobial properties, marking a significant advancement in the authors’ former research. This novel approach produced AgNPs with distinctive structural and biological characteristics for biomedical applications. The findings demonstrated the need for further exploration of green synthesis methods using other extremophile plant species, emphasizing their simplicity, speed, low energy consumption, and eco-friendliness compared with traditional synthesis techniques [13].

The study by Avramescu et al. aimed to evaluate the dissolution behavior of five specific metal oxide-engineered nanomaterials (ENMs)—ZnO, MnO_2_, CeO_2_, Al_2_O_3_, and Fe_2_O_3_—in various aqueous environments, including water and a cell culture medium, at concentrations that are relevant for toxicological research. The researchers also assessed how the particle size influences dissolution by comparing the ENMs with their bulk counterparts. To further understand the solubility dynamics, the dissolution behaviors of ZnO, MnO_2_, and CeO_2_ were examined in two simulated lung fluids, aiming to mimic the conditions of potential human exposure. The study also highlighted that the initial concentration of ENMs is a significant variable to consider when preparing dispersions for toxicological testing [14].

The study by Cifuentes et al. focused on creating innovative nanoengineered extracellular matrix (ECM) scaffolds by combining porcine small intestinal submucosa (SIS) with graphene oxide (GO) and reduced graphene oxide (rGO). With this combination, the authors aimed to enhance electrical conductivity while ensuring excellent biocompatibility. The results demonstrated high hemocompatibility and low cytotoxicity, with no adverse effects on platelet activation and deposition being observed, confirming the scaffolds’ safety for biological applications. Additionally, the structural characteristics of the scaffolds, including their pore size, distribution, and interconnectivity, were optimized to promote effective cell attachment. These features highlight the scaffolds’ potential for diverse applications in regenerative medicine, tissue engineering, and wound healing. By leveraging the unique properties of SIS and graphene derivatives, this research paves the way for advanced biomaterials that can significantly impact healthcare and therapeutic strategies [15].

In conclusion, the two editions of this Special Issue present numerous advancements in the environmental and health applications of ENMs. Each article offers valuable insights and innovative approaches that can enhance our understanding and promote further research in these crucial fields. The contributions highlight the potential of ENMs to address pressing environmental and health challenges. I encourage readers to delve into these articles, as I believe that they will find them not only informative but also instrumental in advancing their research endeavors.
